# How their environment influences endothelial cells

**DOI:** 10.7554/eLife.88248

**Published:** 2023-05-09

**Authors:** Xuejing Liu, Zhen Bouman Chen

**Affiliations:** 1 Arthur Riggs Diabetes Metabolism Research Institute, Beckman Research Institute, City of Hope Duarte United States

**Keywords:** endothelial cell, vascular biology, molecular biology, gene expression, transcriptome, Human

## Abstract

Changes in gene expression in cultured endothelial cells can be partially reversed by simulating in vivo conditions.

**Related research article** Afshar Y, Ma F, Quach A, Jeong A, Sunshine HL, Freitas V, Jami-Alahmadi Y, Helaers R, Li X, Pellegrini M, Wohlschlegel JA, Romanoski CE, Vikkula M, Iruela-Arispe ML. 2023. Transcriptional drifts associated with environmental changes in endothelial cells. *eLife* **12**:e81370. doi: 10.7554/eLife.81370.

Cell culture is a widely used technique in biology (Jensen and Teng, 2020), and cells from the endothelium – the single layer of cells that lines the inside of blood vessels – are often employed in cardiovascular research. Endothelial cells are unique in that they are in direct contact with the blood, and the flow of blood exerts a mechanical force (known as shear stress) that can activate signaling pathways and modify gene expression in the cells. Moreover, endothelial cells are also close to smooth muscle cells (Figure 1), and these two types of cells communicate with each other to maintain healthy blood vessels (Li et al., 2018).

**Figure 1.**
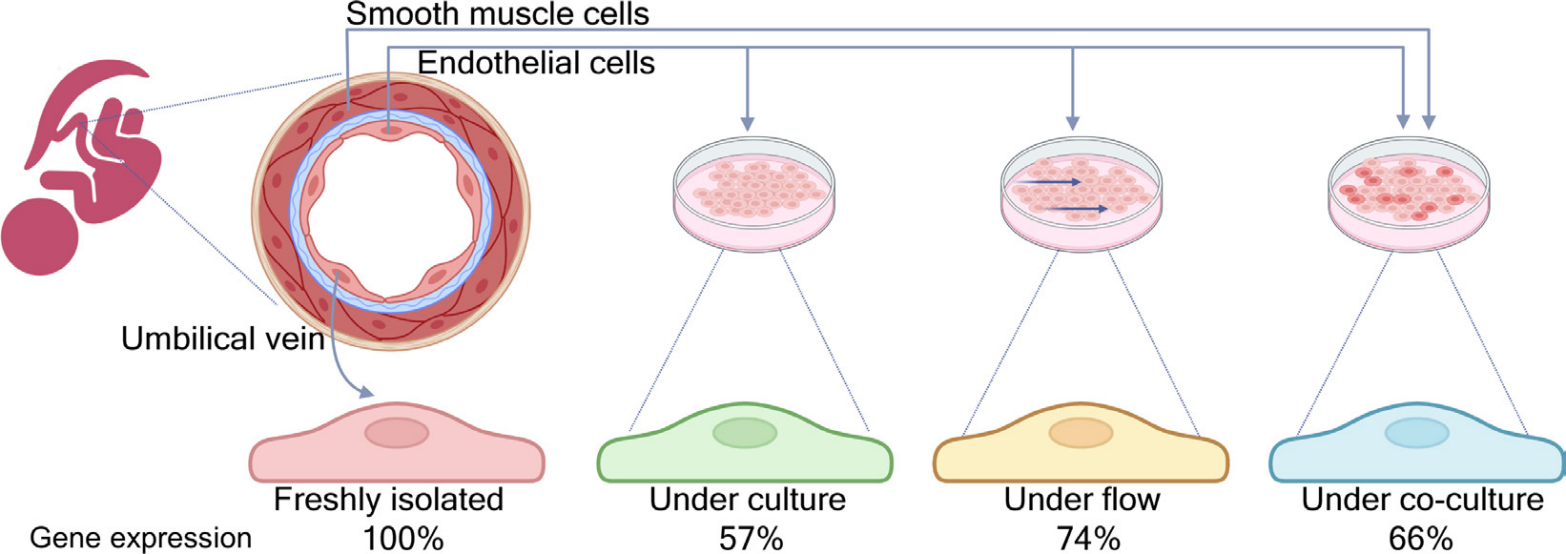
Comparing gene expression profiles in endothelial cells in vivo and under different in vitro conditions. Schematic cross-section of an umbilical vein (top left) made up of a single inner layer of endothelial cells surrounded by a basement membrane (blue) and multiple layers of smooth muscle cells. Afshar et al. compared the gene expression profiles of four samples of endothelial cells: cells freshly isolated from human umbilical cords (left); cord cells cultured in standard conditions; cord cells cultured under flow; and cord cells co-cultured with smooth muscle cells from the same donor (right). Under standard culture conditions, around 57% of the genes expressed in vivo showed no significant changes in vitro. This percentage increased to 74% when the cells were cultured under flow conditions, and to 66% when the endothelial cells were co-cultured with smooth muscle cells.

Image credit: Figure created with BioRender.

Most of the endothelial cells used in cell culture experiments are taken from the umbilical cord of newborn babies, but we do not fully understand how removing these cells from their native environment impacts their behavior and function (Aird, 2007). In particular, it is not clear how the absence of two factors – shear stress and communication between different cell types – changes the behavior of endothelial cells in cell culture experiments. Furthermore, is it possible to change the conditions in cell culture experiments to make them more similar to the conditions in vivo?

Now, in eLife, Luisa Iruela-Arispe (Northwestern University) and colleagues – including Yalda Afshar (University of California) as first author – report that gene expression in endothelial cells is altered when the cells are cultured in vitro, and can be partially restored if the cells are exposed to shear stress and smooth muscle cells (Afshar et al., 2023).

Using endothelial cells taken from the umbilical cords of seven human donors, Afshar et al. compared the gene expression profiles of cells cultured under in vitro conditions with the profiles of cells freshly isolated from the same donor (hereafter called cord cells). RNA sequencing showed that nearly half of the genes expressed in the culture condition were different from those expressed in the freshly isolated cells: in particular, genes sensitive to blood flow were expressed less, and genes related to cell proliferation were expressed more (Figure 1).

Afshar et al. then modified the culture conditions to see if the gene expression profile could be made more similar to the profile of the cord cells. First, the cultured cells were placed under flow conditions for 48 hours to simulate the shear stress caused by blood flow (Chiu and Chien, 2011). This led to the expression of a number of genes that were not expressed in culture without flow, including genes which belong to two signaling pathways – BMP and NOTCH – that are known to be sensitive to flow (Souilhol et al., 2020).

Next, Afshar et al. co-cultured endothelial cells alongside smooth muscle cells from the same donor. Single-cell RNA sequencing showed that this restored the expression of various genes (including genes for cytoskeleton proteins) that were not expressed when endothelial cells were cultured on their own. Moreover, the expression of genes related to cell proliferation was reduced. Furthermore, the team also built “Flow Profiler”, an open-source website to display the analyzed datasets and allow further exploration of the behavior of genes under flow conditions.

The results of Afshar et al. demonstrate the impact of culture conditions on gene expression in a systematic and quantitative fashion, and highlight the importance of contextual information when interpreting experimental results. Going forward it would be interesting to explore if introducing flow and smooth muscle cells at the same time would restore even more of the gene expression profile. Furthermore, endothelial cells are found in a variety of vessels in the body, and the flow pattern (and hence the shear stress) will be different in different vessels: future work could explore the impact of different locations and flow patterns on gene expression profiles. It would also be interesting to investigate the influence of DNA methylation and histone modification on gene expression in endothelial cells. The knowledge and insights gained from such studies will help researchers to develop more representative cell culture models for the study of cells and organs.

Researchers are also exploring a range of other techniques that mimic the in vivo environment, such as 3D bioprinting (Neufeld et al., 2022), organoids (LeSavage et al., 2022) and organ-on-a-chip technologies (Tolabi et al., 2023). Combining all these techniques, including improved cell culture models, should lead to a better understanding of cell behavior and disease mechanisms, and thus help researchers working to improve medical outcomes in scenarios where endothelial function is impaired.
